# Supplementary figures and images for: Transcriptomic and proteomic intra-tumor heterogeneity of colorectal cancer varies depending on tumor location within the colorectum
Source: PLoS One. 2020 Dec 17;15(12):e0241148. doi: 10.1371/journal.pone.0241148 (PMC7746197; doi:10.1371/journal.pone.0241148)

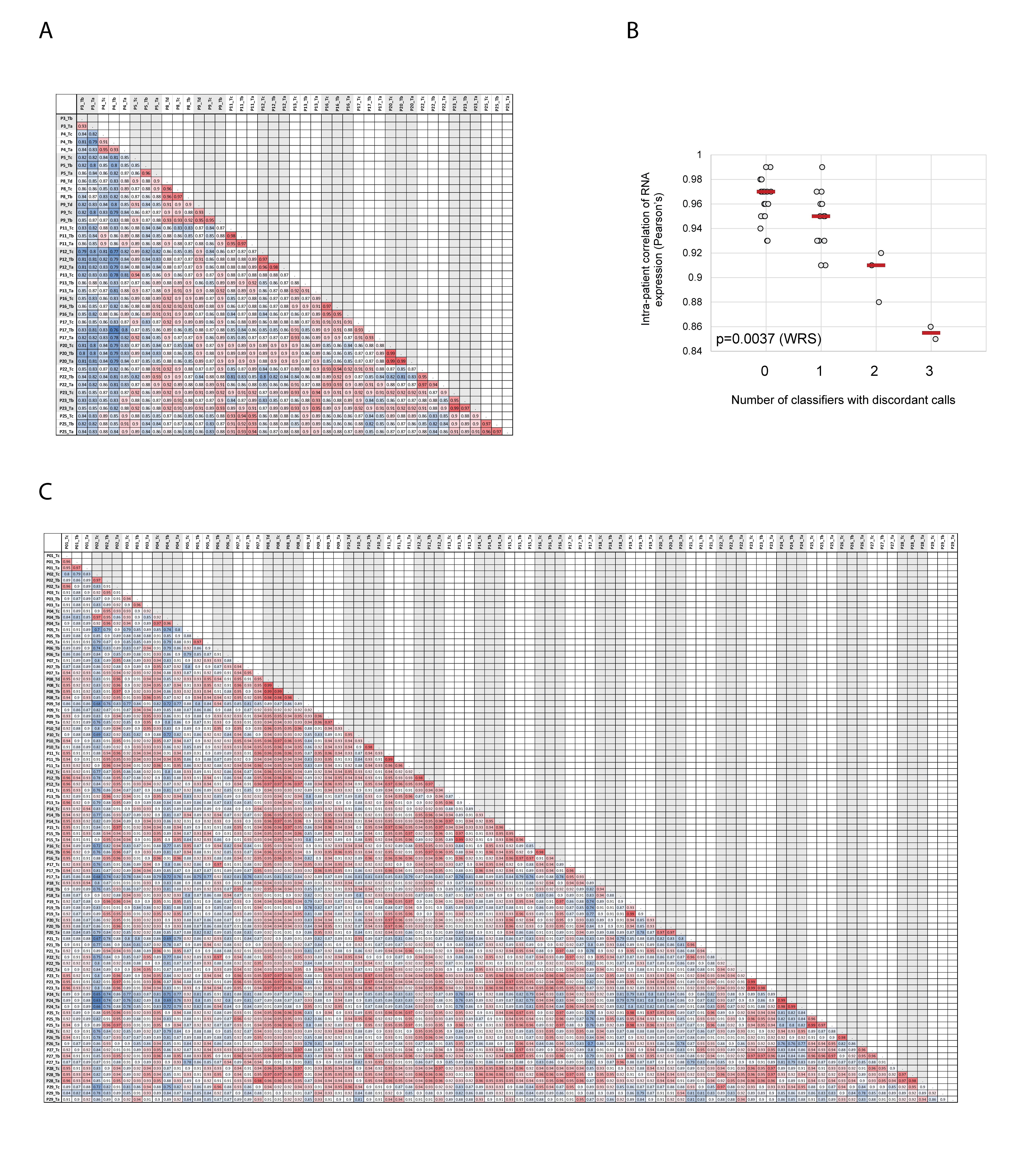

Supplement: S1 Fig — (A) Correlation matrix showing the pair-wise, inter-sample correlations (Pearson’s r) in RNA expression profiles (12,593 RNAs with average expression >1 (log2(CPM))). (B) Plot showing the intra-tumor correlation in RNA expression between biopsies (Pearson’s r; Y-axis) according to the number of classifiers (CMS, CRIS, TT) that exhibit discordant subtype calls for a tumor sample (X-axis; no discordant calls is ‘0’, whereas values 1, 2 and 3 indicate the number of classifiers that exhibit discordant subtype calls for a tumor). Biopsies that have discordant calls exhibits lower correlation in RNA expression to the other biopsies from the same tumor than biopsies with no discordant calls. Red bar indicate average values for each category. The p-value indicates that biopsies with no discordant calls for any classifier (X-axis = 0) exhibit significantly higher intra-tumor correlation in RNA expression than biopsies from tumors with discordant classifier calls (X-axis = 1–3) as evaluated by a Wilcoxon rank sum test (WRS). (C) Correlation matrix showing the pair-wise, inter-sample correlations (Pearson’s r) in protein expression profiles (68 proteins). (TIF) [file pone.0241148.s001.tif]

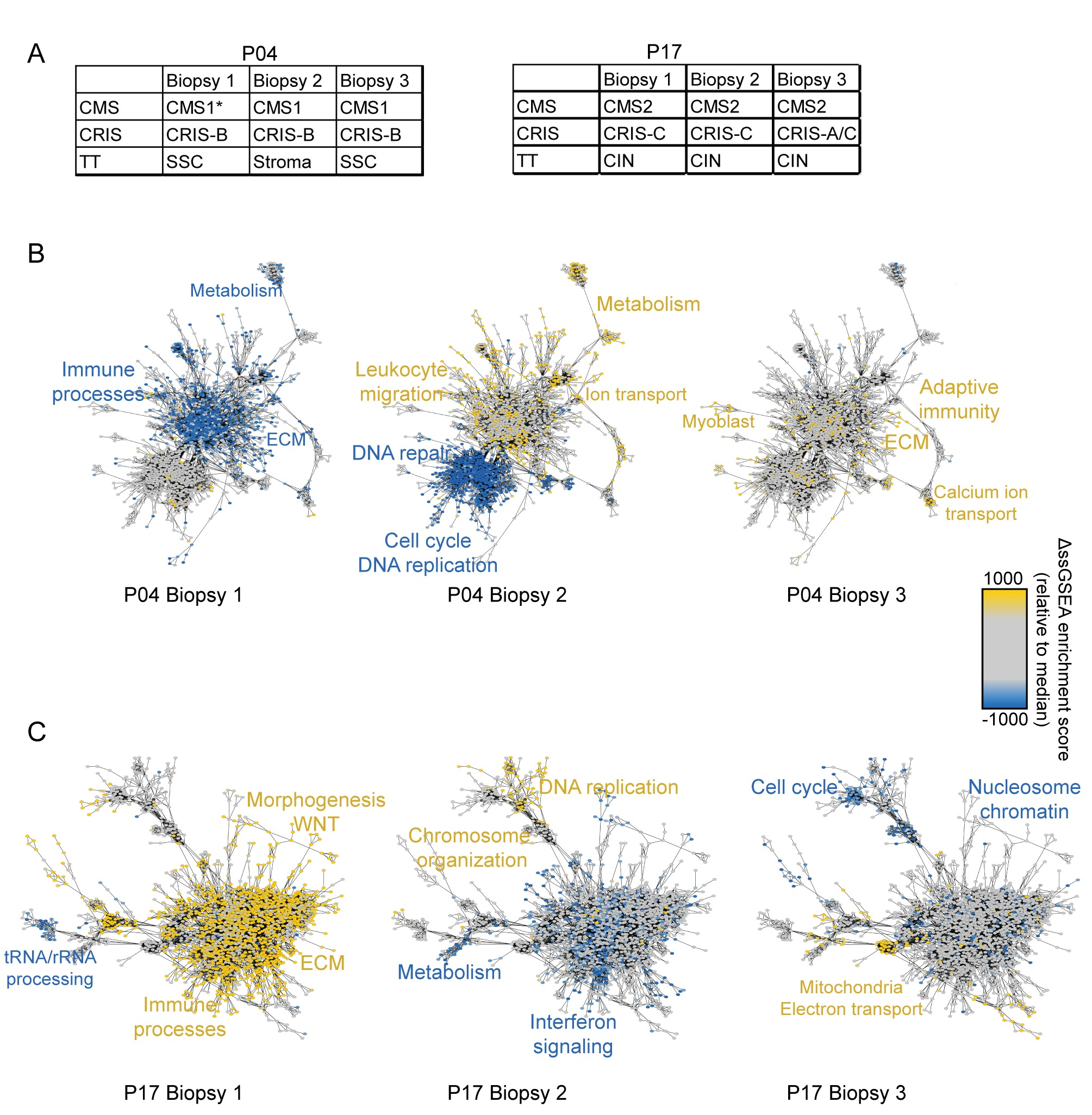

Supplement: S2 Fig — (A) Subtyping results for each biopsy from tumors P04 and P17. (B-C) Tumor-specific network maps for two tumors illustrating the 5000 ssGSEA terms with the highest ITH for tumor P04 in (B) and tumor P17 in (D). Yellow dots/font indicates mechanisms that are upregulated in the sample compared to the other samples from the same tumor, while blue indicates downregulated mechanisms. (TIF) [file pone.0241148.s002.tif]
